# Preclinical Evaluation of the Immunomodulatory Properties of Cardiac Adipose Tissue Progenitor Cells Using Umbilical Cord Blood Mesenchymal Stem Cells: A Direct Comparative Study

**DOI:** 10.1155/2015/439808

**Published:** 2015-03-10

**Authors:** Isaac Perea-Gil, Marta Monguió-Tortajada, Carolina Gálvez-Montón, Antoni Bayes-Genis, Francesc E. Borràs, Santiago Roura

**Affiliations:** ^1^ICREC Research Program, Germans Trias i Pujol Health Science Research Institute, Can Ruti Campus, 08916 Badalona, Spain; ^2^IVECAT Group, Germans Trias i Pujol Health Science Research Institute, Can Ruti Campus, 08916 Badalona, Spain; ^3^Cardiology Service, Germans Trias i Pujol University Hospital, 08916 Badalona, Spain; ^4^Department of Medicine, UAB, 08916 Badalona, Spain; ^5^Nephrology Service, Germans Trias i Pujol University Hospital, 08916 Badalona, Spain

## Abstract

Cell-based strategies to regenerate injured myocardial tissue have emerged over the past decade, but the optimum cell type is still under scrutiny. In this context, human adult epicardial fat surrounding the heart has been characterized as a reservoir of mesenchymal-like progenitor cells (cardiac ATDPCs) with potential clinical benefits. However, additional data on the possibility that these cells could trigger a deleterious immune response following implantation are needed. Thus, in the presented study, we took advantage of the well-established low immunogenicity of umbilical cord blood-derived mesenchymal stem cells (UCBMSCs) to comparatively assess the immunomodulatory properties of cardiac ATDPCs in an *in vitro* allostimulatory assay using allogeneic mature monocyte-derived dendritic cells (MDDCs). Similar to UCBMSCs, increasing amounts of seeded cardiac ATDPCs suppressed the alloproliferation of T cells in a dose-dependent manner. Secretion of proinflammatory cytokines (IL6, TNF*α*, and IFN*γ*) was also specifically modulated by the different numbers of cardiac ATDPCs cocultured. In summary, we show that cardiac ATDPCs abrogate T cell alloproliferation upon stimulation with allogeneic mature MDDCs, suggesting that they could further regulate a possible harmful immune response *in vivo*. Additionally, UCBMSCs can be considered as valuable tools to preclinically predict the immunogenicity of prospective regenerative cells.

## 1. Introduction

The use of multipotent “stem” or progenitor cells in multiple clinical settings is rather encouraging [1–3]. This is, for instance, the case of interest in the regenerative ability of the human heart following acute myocardial infarction, which overloads the functional capacity of the surviving myocardium and ultimately leads to heart failure [4]. To date, the unique treatment that fully restores lost cardiac function after advanced heart failure is heart transplantation. Cell-based strategies to regenerate myocardial tissue have emerged over the past decade, but the optimum cell type is still under scrutiny. Although the existence of resident cardiac stem cells has been reported [5, 6], their capacity of tissue repair after damage is scarce; thus, one of the main objectives in this field of research is to find the best cell type for repairing injured cardiac tissue [7].

A population of human adult mesenchymal-like progenitor cells derived from cardiac adipose tissue (cardiac ATDPCs), with inherent cardiac and endothelial cell potential, was isolated and characterized for the first time in our laboratory [8]. Taken together, our results showed that cardiac ATDPCs could play a role in heart homeostasis, perhaps as a cell reservoir for renewing myocardial tissue. Based on these findings, we are planning to investigate the potential clinical benefits of these cells. However, additional data on the possibility that they could elicit a deleterious antidonor immune response are needed.

In this context, recent data have showed that mesenchymal stem cells (MSCs) derived from umbilical cord blood (UCB) have low immunogenicity and are, therefore, immunologically safe for use in allogeneic clinical applications [9]. The lower immunogenicity of UCBMSCs is attributed to its immaturity, in contrast to alternative adult cell sources. Yet, not all adult cell sources have been characterized regarding their immunogenicity.

Thus, in the presented study, we took advantage of the well-established low immunogenicity of UCBMSCs to comparatively assess the immunomodulatory properties of cardiac ATDPCs in an* in vitro* allostimulatory assay.

## 2. Materials and Methods

### 2.1. Cardiac ATDPC and UCBMSC Isolation and Culture

The study protocols were approved by the Clinical Research Ethics Committee of our institution (Comitè Ètic d'Investigació Clínica, HuGTiP, Refs. CEIC: EO-10-13, EO-10-016, and EO-12-022) and conformed to the principles outlined in the Declaration of Helsinki. Written informed consent was obtained from donors.

Cardiac ATDPCs were extracted from adipose tissue surrounding the base of the heart and around the aortic root from patients undergoing cardiothoracic surgery prior to cardiopulmonary bypass initiation, as reported in [8]. Adipose tissue specimens were washed in sterile PBS (Invitrogen, Carlsbad, CA) to remove red blood cell contamination and treated with a 0.05% collagenase type-II solution (Gibco Life Technologies/Invitrogen, Carlsbad, CA) for 30 min at 37°C in gentle agitation. Cell suspension was centrifuged at 1200 ×g for 10 min, resuspended in complete medium corresponding to *α*-MEM (Sigma Aldrich, St. Louis, MO) supplemented with 10% heat-inactivated FBS, 2 mM L-glutamine (Gibco), 1% penicillin/streptomycin, and 5 *μ*g/mL Plasmocin (Invivogen, San Diego, CA), and grown under standard culture conditions.

UCBMSCs were isolated and cultured as described previously [10–12]. In brief, UCB samples were collected from the umbilical cord vein and processed within 12 hours after extraction. Blood was clarified by centrifugation, and blood cells were resuspended in magnesium- and calcium-free PBS (Invitrogen). The cell suspension was then layered over 1.077 g/mL Lymphoprep (Axis-Shield, Oslo, Norway), and mononuclear cells (MNCs) were recovered by centrifugation at 400 ×g for 30 min. Red blood cells were eliminated after incubation with Lysis Reagent Pharm-Lyse (BD Biosciences) for 15 min. Adherent cells were cultured in complete *α*-MEM under standard culture conditions.

### 2.2. Monocyte-Derived Dendritic Cell Generation

Culture media were composed of RMPI 1640 (Gibco Life Technologies/Invitrogen) supplemented with L-glutamine (Sigma Aldrich, St. Louis, MO), 100 U/mL penicillin (Cepa S.L., Madrid, Spain), 100 *μ*g/mL streptomycin (Normon Laboratories S.A., Madrid, Spain), and 5% (v/v) human serum AB (BioWhittaker/Lonza, Basel, Switzerland).

Peripheral blood MNCs were obtained from leukocyte residues from healthy donors from the Blood and Tissue Bank (Barcelona, Spain) by Ficoll Hypaque Plus density gradient centrifugation (GE Healthcare Biosciences, Uppsala, Sweden) at 1800 rpm for 30 minutes. Monocytes were then isolated using the EasySep Human Anti-CD14 Positive Selection Kit (StemCell Technologies, Grenoble, France), and recovered cells were counted using PerfectCount Microspheres (Cytognos, Salamanca, Spain). The purity obtained was >90% and viability ≥93% FSC/SSC gating. Monocytes were then either differentiated to dendritic cells or kept frozen until use.

In particular, monocyte-derived dendritic cells (MDDCs) were generated by plating 1 · 10^6^ monocytes/mL in complete medium supplemented with the dendritic cell differentiation cytokines 300 IU/mL IL4 and 450 IU/mL GM-CSF (both from Miltenyi Bitech, Bergisch Gladbach, Germany). Cell feeding was performed every two days and the maturation stimulus LPS (500 ng/mL, Sigma Aldrich) was added at day 5. Mature MDDCs were obtained at day 6 after washing with PBS (Thermo Scientific HyClone, Logan, UT, USA) and detaching cells with accutase (eBioscience, San Diego, CA) for 30 minutes at 37°C. Cells were then counted using PerfectCount Microspheres (Cytognos) obtaining a >80% viability.

### 2.3. Allostimulatory Assays

For these experiments, peripheral blood MNCs from leukocytes residues from healthy donors (Blood and Tissue Bank) were also obtained by Ficoll Hypaque Plus (GE Healthcare) density centrifugation. T cells were then isolated from peripheral blood MNCs using the EasySep Human T cell Enrichment Kit (StemCell Technologies) following manufacturer's instructions. Briefly, cells were diluted in column buffer at a concentration of 5 · 10^7^ cells/mL, labeled with the Tetrameric Antibody Complexes (TAC) recognizing human CD14, CD16, CD19, CD20, CD36, CD56, CD66b, CD123, glycophorin A, and dextran-coated magnetic particles, and separated using the EasySep magnet (StemCell Technologies). Enriched T cells were then washed and stained with carboxyfluorescein diacetate succinimidyl ester (CFSE) (Molecular Probes, Leiden, Netherlands) to assess cell proliferation as described previously [13]. Briefly, enriched T cells were resuspended in PBS for staining with an equal volume of 0.8 *μ*M CFSE. After 10 minutes, labeled T cells were washed twice with RPMI (Sigma Aldrich) supplemented with 10% FBS and plated at 3 · 10^5^ cells/well in flat-bottomed well plates in which allogeneic cardiac ATDPCs or UCBMSCs (20.000, 10.000, 5.000, 2.500, or 1.250 cells/well) had been previously seeded. T cells had a viability of over 94% in all experiments performed.

After an overnight resting period, allogeneic mature MDDCs were added at either 20 : 1 or 40 : 1 T cell : MDDC ratios to the indicated wells. Alloproliferation was determined after 4.5 days by measuring the CFSE^low/neg^ population by flow cytometry in a LSR Fortessa Analyzer (BD Biosciences). Results were reported as the mean % FSC^high^CFDA-SE^low^ population ± SD in triplicate culture wells relative to the 20 : 1 ratio with 1250 MSCs seeded.

### 2.4. Measurement of Cytokine Production

Cytokines present in supernatants from alloproliferation assays collected at day 4.5 were measured using the CBA human Th1/Th2 Cytokine kit II (BD Biosciences). Concentrations of IL2, IL4, IL6, IL10, and TNF*α* were assessed following manufacturer's instructions in a LSR Fortessa Analyzer (BD). The minimum detectable concentration (pg/mL) of each protein was 2.6 for IL2 and IL4, 3.0 for IL6, and 2.8 for IL10 and TNF*α*. All the cytokines measured exceeded the detection limit mentioned before.

### 2.5. Statistical Analysis

Values are expressed as mean ± standard deviation (SD). Statistical analysis was performed using two-tailed Student's* t*-tests when two groups were compared, and the Greenhouse-Geisser method for repeated measures was applied to determine significance between two groups with different cell number data. All analyses were performed with SPSS statistic software (19.0.1 version, SPSS Inc., Chicago, IL), and differences were considered significant when *P* < 0.05.

## 3. Results

### 3.1. Cardiac ATDPCs Reduce the Alloproliferative Response of T Cells

**Figure 1:**
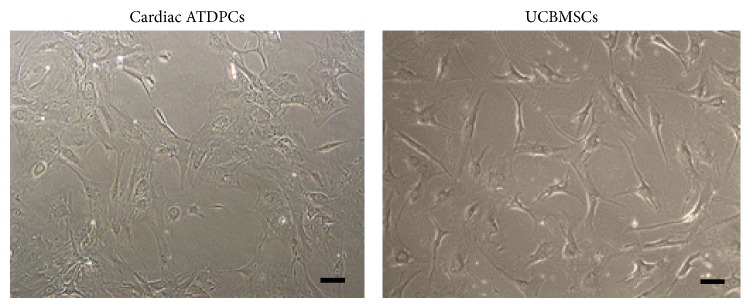
Cardiac ATDPC and UCBMSC cultures. Representative phase-contrast images of cardiac ATDPCs and UCBMSCs grown in standard conditions of* in vitro* culture. Scale bars = 100 *μ*m.

In these experiments, we sought to compare the immunomodulatory properties of the uncharacterized cardiac ATDPCs (Figure 1(a)) with the well-established nonimmunogenic UCBMSCs (Figure 1(b)). Cardiac ATDPCs had originally been characterized as MSC-like cell progenitors, with over 90% of cells staining strongly positive for CD105, CD44, CD166, CD29, and CD90 and negative for CD106, CD45, and CD14 [8]. Interestingly, their culture in adipogenic differentiation media did not result in intracellular accumulation of lipid droplets [8]. Moreover, primary cultures of elongated fibroblast-like cells established from UCB had strictly been homogeneous and previously recognized as MSCs by our group [10, 12]. These cell cultures were consistently positive for CD105, CD44, CD166, CD29, and CD90 and negative for CD117, CD106, CD34, CD45, CD14, VEGF-R2, and CD133 as well as differentiated into adipogenic, chondrogenic, and osteogenic cell lineages under specific conditions.

**Figure 2:**
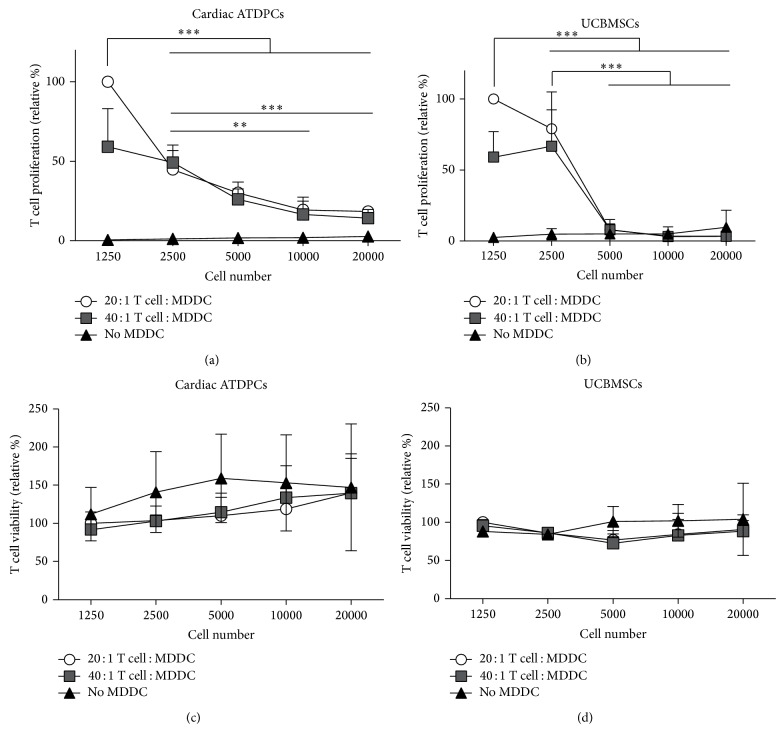
T cell alloproliferation induced by MDDCs is abrogated with either cardiac ATDPCs or UCBMSCs. Proliferation was calculated as % of FSC^high^CFSE^low^ T cells after a 4.5-day coculture with allogeneic mature MDDCs in 20 : 1 and 40 : 1 ratios, together with different numbers (1,250, 2,500, 5,000, 10,000, and 20,000 cells) of either cardiac ATDPCs (a) or UCBMSCs (b). Viability of T cells was assessed according to FSC/SSC gating in coculture with cardiac ATDPCs (c) and UCBMSCs (d). Results are reported as % of proliferation or viability relative to the proliferation or viability level of T cells stimulated with MDDCs in a 20 : 1 ratio, respectively. Data is depicted as mean ± SD of four independent experiments (*n* = 4 for UCBMSCs, cardiac ATDPCs, and responder T cells), calculated from three experimental replicates. Statistical differences, indicated as ^**^ *P* < 0.01 and ^***^ *P* < 0.001, are only shown for the 20 : 1 T cell : MDDC ratio.

T cell proliferation was induced by allogeneic mature MDDCs at two different T cell : MDDC ratios (20 : 1 and 40 : 1) in the presence of increasing numbers of either cardiac ATDPCs or UCBMSCs, both from a third party donor. As shown in Figure 2(a), increasing amounts of seeded cardiac ATDPCs significantly suppressed the alloproliferation of T cells in a dose-dependent manner (*P* < 0.001 for both 20 : 1 and 40 : 1 ratios). Similarly, UCBMSCs also significantly suppressed the induced alloproliferation of T cells (*P* < 0.001 for both 20 : 1 and 40 : 1 ratios) (Figure 2(b)). However, a minimum amount of seeded UCBMSCs (5,000 cells or higher) was able to drastically abrogate T cell alloproliferation, fully reaching the values of the negative control (no MDDC stimulation), while lower numbers did not show a clear immunomodulatory effect. Importantly, this effect was observed at the two T cell : MDDC ratios studied (*P* < 0.001 comparing 2,500 with higher UCBMSC numbers, for both ratios).

Remarkably, the lower proliferation levels of T cells cocultured with cardiac ATDPCs or UCBMSCs were not related to the induction of cell death, as the viability of cultured T cells was at least maintained in all conditions tested (Figures 2(c) and 2(d)). In fact, T cell viability was actually higher in cells cocultured with increasing numbers of cardiac ATDPCs, although the values did not reach statistical significance (*P* = 0.371, *P* = 0.238, and *P* = 0.835 for 20 : 1 ratio, 40 : 1 ratio, and without MDDCs, resp.) (Figure 2(c)). This would indicate a feeding-like effect of cardiac ATDPCs on allogeneic T cells, promoting cell viability while minimizing immune response to alloantigens.

**Figure 3:**
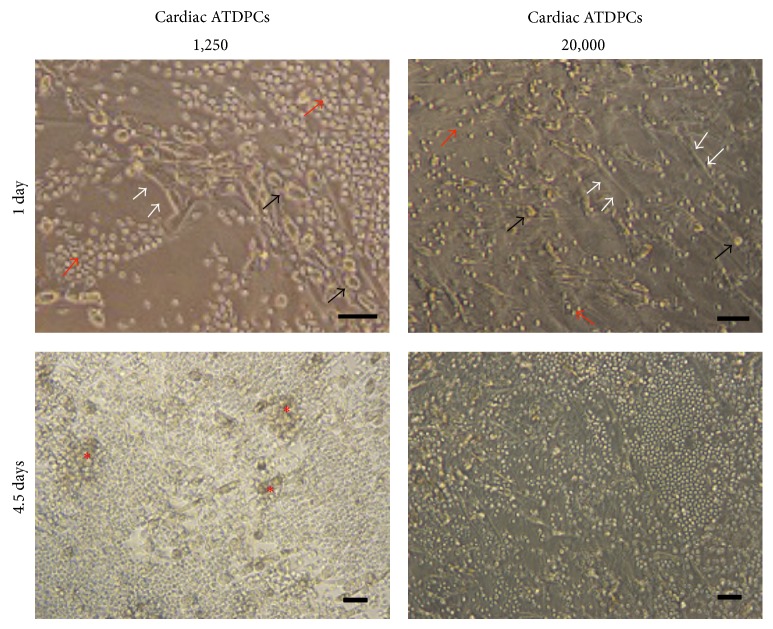
Formation of T cell proliferation clusters is affected by increasing numbers of cardiac ATDPCs in the allostimulatory assay. Representative phase-contrast images of 1,250 and 20,000 cardiac ATDPCs (white arrows) cocultured along with T cells (red arrows) and allogeneic mature MDDCs (black arrows) at 1 and 4.5 days. Red asterisks indicate T cell proliferation clusters at 4.5 days. Similar images were taken from UCBMSC cocultures. Scale bars = 100 *μ*m.

Figure 3 depicts some images of the cell culture experiments showing the three types of cell partners and the proliferative cell clusters induced by allogeneic mature MDDCs in the presence of low numbers of cardiac ATDPCs and UCBMSCs.

### 3.2. Modulation of Cytokine Secretion during Allostimulation in the Presence of Cardiac ATDPCs and UCBMSCs

**Figure 4:**
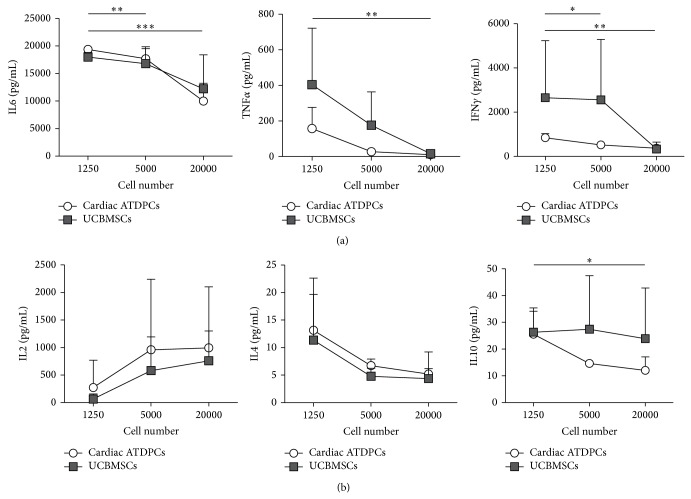
Cytokine levels are specifically modulated by the number of cardiac ATDPCs or UCBMSCs seeded in the allostimulatory assay. Histograms represent detected levels of early stage (a) and late stage (b) cytokines in the supernatants from cocultures of increasing numbers of cardiac ATDPCs (white circles) or UCBMSCs (black squares) with T cells and allogeneic mature MDDCs (20 : 1 ratio). Data are expressed as mean ± SD from four independent experiments (*n* = 4 for UCBMSCs, cardiac ATDPCs, and responder T cells). Statistical differences, indicated as ^*^ *P* < 0.05, ^**^ *P* < 0.01, and ^***^ *P* < 0.001, were only found for cardiac ATDPCs.

To further compare the immunomodulation ability of cardiac ATDPCs, the production of several cytokines was assessed in the supernatants from T cell cocultures with allogeneic mature MDDCs (20 : 1 T cell : MDDC ratio) in the presence of different numbers of cardiac ATDPCs or UCBMSCs (1,250, 5,000, or 20,000). The secretion of proinflammatory cytokines during allostimulation, including IL6, TNF*α*, and IFN*γ*, was significantly decreased when experiments were performed in the presence of increasing numbers of cardiac ATDPCs (Figure 4(a)). Although the levels of TNF*α* and IFN*γ* were slightly higher in alloproliferation assays cocultured with low numbers of UCBMSCs compared to cardiac ATDPCs, the results obtained were not significantly different (for 1,250 cells, *P* = 0.195 and *P* = 0.256, for TNF*α* and IFN*γ*, resp.). Regarding IL6, the levels were almost identical in allostimulatory cocultures with both cardiac ATDPCs and UCBMSCs. In particular, cardiac ATDPCs showed an important reduction of IL6 production (about 50%) when 20,000 cells were seeded compared to 1,250 cells (*P* = 0.001). Other cytokines analyzed were IL2, IL4, and IL10 (Figure 4(b)). A modest increase in IL2 levels was observed when increasing numbers of both tested cell populations had been seeded, but no statistical difference was reached (*P* = 0.157 and *P* = 0.552 for cardiac ATDPCs and UCBMSCs, resp.). In contrast, IL4 secretion was equally reduced with the presence of increasing amounts of both cardiac ATDPCs and UCBMSCs. Regarding IL10, their levels were significantly reduced with increasing numbers of cardiac ATDPCs (*P* = 0.021). Conversely, UCBMSCs cultures did not show a clear reduction of IL10 levels.

## 4. Discussion

Researchers concur with the idea that the optimal source of cells for cardiac regeneration should (1) be autologous, in order to reduce severe complications of the immune system and disease transmission; (2) exhibit a controlled cell division capacity; (3) differentiate towards both cardiomyogenic and endothelial cell lineages; and (4) integrate efficiently and functionally into injured myocardium after cell implantation. Although considerable efforts have been made, the finding of the cell type with the best regenerative potential remains a challenge [14]. In this context, we focused our attention on cardiac adipose tissue [15], identifying a progenitor cell population resident in the adult human cardiac adipose tissue, which showed potential to differentiate into cardiac and endothelial cell lineages* in vitro* and exhibit beneficial effects upon transplantation in experimental models of myocardial infarction in rodents, promoting angiogenesis in the ischemic tissue [8]. This cell population also expressed MSC-like surface markers but, unlike bona fide MSCs such as those derived from subcutaneous adipose tissue, showed less plasticity as evaluated by flow cytometry and in adipogenic differentiation assays [8]. Therefore we concluded that, although residing in an adipocytic environment, cardiac ATDPCs show a committed condition to cardiac-like phenotype.

Regarding immunoreactivity, as an exception, allogeneic MSCs are also being considered for tissue regeneration due to their inherent immunoprivileged features. Particularly, MSCs show low expression of major histocompatibility complex (MHC) class I and lack of class II MHC in resting conditions and/or costimulatory molecules, such as CD40, CD80, or CD86 [16, 17]. Moreover, they are characterized to have immunomodulatory effects via the inhibition of both B and T cell proliferation, promoting allograft survival and reducing graft versus host disease [18–25]. However, other studies have suggested that MSCs are immunogenic and promote the immune response, as infusions of allogeneic MSCs were found to foster the rejection of skin allografts and exacerbate T cell proliferation towards the infused MSCs themselves, leading to the generation of functional memory T cells [26–29]. Collectively, these controversial results point out different immunological outcomes of infused MSCs depending on their activated state [24, 30], and reinforce the importance of more preclinical studies and screening of MSC immunoreactivity before their clinical use.

With this goal, we focused on the immunophenotyping of the uncharacterized human adult mesenchymal-like progenitor cells derived from cardiac adipose tissue (cardiac ATDPCs). For this purpose, we compared these cells to the already well-studied nonimmunogenic human UCBMSCs [9]. UCB is considered the most plentiful reservoir of regenerative cells for a variety of clinical applications [31, 32]. Although used mainly against blood disorders such as blood malignancies and immune deficiencies, the spectrum of diseases for which UCB provides helpful treatment has been expanded to nonhematopoietic conditions, including cell-based therapy and immunomodulation [33]. Despite MSCs were sought to be present in UCB at a low frequency in contrast to their presence in other tissue sources, such as bone marrow, adipose tissue, or umbilical cord, transplantation of double partially HLA-matched UCB units is a simple approach for overcoming this important limitation [33, 34]. Moreover, procedures for UCBMSCs' isolation have been enhanced along the last years. Importantly, recent work demonstrates that MSCs can be expanded successfully from 30% to 60% of low-volume UCB units [35]. In terms of advantages, in contrast to other perinatal stem cell sources such as umbilical cord, establishment of primary UCBMSC cultures does not lead to a relatively high-cost and long procedure based in enzymatic digestion or explant methods. UCB can also be safe and painlessly extracted, long-term cryopreserved, and has a lower risk of transmitting viral infections or somatic mutations than adult tissues (i.e., bone marrow). In line, we also recognize UCBMSCs as a useful cellular population to preclinically assess the immunoreactivity of prospective therapeutic cells.

Thus, in the presented study, we demonstrate for the first time that cardiac ATDPCs also exert a great immunosuppression activity because increasing numbers of these cells abrogate T cell proliferation in a coculture setting using third party mature MDDCs [36]. These findings are in line with previous studies indicating that adipose tissue-derived multipotent stromal cells were more active in reducing T cell proliferation at lower cell ratios than their bone marrow-derived counterparts [18–20, 37–39]. Importantly, this behavior of cardiac ATDPCs is not significantly different from that exhibited by the well-described immunosuppressive UCBMSCs. However, some differences arise from the comparative immunomodulatory analysis of the two cellular populations tested. The antiproliferative effect of cardiac ATDPCs is dose-dependent in comparison with that exerted by UCBMSCs. Apparently, seeding a minimum amount of UCBMSCs is able to drastically abrogate T cell alloproliferation. This result may indicate a stronger immunoregulatory capacity of UCBMSCs, probably attributed to their immature characteristics.

We further show that proinflammatory cytokine secretion in alloproliferation assays is specifically modulated by the different numbers of cardiac ATDPCs cocultured. In particular, the low levels of early stage cytokines (IL6, TNF*α*, and IFN*γ*) would correlate with the immunosuppressive effect seen when analyzing the proliferation assays. Remarkably, in an* in vivo* scenario, the induction of low on-site inflammation would diminish the probabilities of effector cell recruitment towards implanted cells. In addition, the analysis of the production of late stage cytokines (IL2, IL4, and IL10) revealed a modest increase in IL2 levels when increasing numbers of both cardiac ATDPCs and UCBMSCs had been seeded. These results may correlate with the sustained viability of T cells cocultured with both cell types, as seen before for other MSC sources [40], and ruling out the possibility of reduced T cell proliferation due to cell death induction [38, 39].

Our results also suggest that cardiac ATDPCs would not be responsible for the proinflammatory state in patients with heart failure and atherosclerosis [41]. Human epicardial fat surrounding the heart, which consists of adipocytes and a stromal fraction, is an active source of multiple bioactive molecules that substantially influence the myocardium and coronary arteries [15]. Moreover, expression levels of the anti-inflammatory molecule adiponectin are shown to be significantly reduced in epicardial fat in comparison with subcutaneous adipose tissue [42]. However, as our findings appear to indicate, the stromal fraction of epicardial fat containing the cardiac ATDPC population may confer some protective effects counteracting its well-described proinflammatory activity.

In summary, the main finding of the present study is that cardiac ATDPCs abrogate the alloproliferation of T cells upon stimulation with allogeneic mature MDDCs, suggesting that they could further regulate a possible harmful antidonor immune response following* in vivo* implantation [43]. Additionally, we suggest that UCBMSCs can be considered as valuable tools to preclinically predict the immunogenicity of prospective regenerative cells.
